# Self-Healing Antimicrobial Silicones—Mechanisms and Applications

**DOI:** 10.3390/polym15193945

**Published:** 2023-09-29

**Authors:** Anna Kowalewska, Kamila Majewska-Smolarek

**Affiliations:** Centre of Molecular and Macromolecular Studies, Polish Academy of Sciences, Sienkiewicza 112, 90-363 Lodz, Poland; kamila.majewska-smolarek@cbmm.lodz.pl

**Keywords:** self-healing, silicones, polysiloxane, PDMS, antimicrobial, antibacterial, antifouling

## Abstract

Organosilicon polymers (silicones) are an important part of material chemistry and a well-established commercial product segment with a wide range of applications. Silicones are of enduring interest due to their unique properties and utility. Recently, new application areas for silicone-based materials have emerged, such as stretchable electronics, wearable stress sensors, smart coatings, and soft robotics. For this reason, research interest over the past decade has been directed towards new methods of crosslinking and increasing the mechanical strength of polyorganosiloxanes. The introduction of self-healing mechanisms may be a promising alternative for such high-value materials. This approach has gained both growing research interest and a rapidly expanding range of applications. Inherent extrinsic and intrinsic self-healing methods have been used in the self-healing of silicones and have resulted in significant advances in polymer composites and coatings, including multicomponent systems. In this review, we present a summary of research work dedicated to the synthesis and applications of self-healing hybrid materials containing polysiloxane segments, with a focus on antimicrobial and antifouling coatings.

## 1. Introduction

Polymers made of siloxane bonds (silicones: polysiloxanes and polysilsesquioxanes) are recognized high-value materials of excellent low-temperature flexibility, high and low-temperature stability, low surface energy, high permeability, electrical resistance, high oxidation stability, and resistance to environmental conditions, as well as biocompatibility, sterilization tolerance, biological durability, and hemocompatibility [1,2,3,4]. Owing to their intrinsic properties, silicones can be used not only in the electronics or aerospace industry but also in the biomedical field and the food industry, where durability, sterility, and chemical purity of polymeric materials are required. Poly(dimethylsiloxane) (PDMS) is the most well-known example of silicone-based materials, a precursor of covalently cross-linked elastomers that are soft, stretchable, and almost creep-free. The mechanical performance of siloxane polymers can be tuned by adjusting their molecular weight or degree of cross-linking. However, conventional chemical cross-linking does not guarantee durability, and thus the main disadvantage of traditional silicone elastomers is their susceptibility to physical damage.

While silicone materials of this type are still in high demand, modern technologies, much more oriented towards sustainability, recyclability, and reusability, tend to focus on the dynamic crosslinking of siloxanes with tailored mechanical properties by means of self-healing (S-H). The introduction of a S-H mechanism into silicone materials may be a promising alternative to covalent network formation, and self-regenerating polysiloxane elastomers that are capable of healing minor physical damage are the focus of recent research [5,6,7,8,9]. The self-repair capability can not only extend the life of silicone coatings but also help meet the specific requirements of emerging new applications such as stretchable electronics, wearable stress sensors, smart coatings, and soft robotics.

An important concern for materials intended for such applications, aside from their self-healing effectiveness and excellent mechanical properties, is their resistance to biological factors. Frequent and prolonged contact with skin, moisture, and the external environment makes each type of silicone material susceptible to unwanted microbial deposition and biofilm growth. Any major damage but also any small changes in the continuity of polymer coatings (e.g., minor scratches) can create favorable conditions for biofilm formation and have a huge impact on the rate of surface colonization by microorganisms. Scratches can become specific habitats—protective environmental niches for bacterial growth. In this context, the capability for effective re-mending and, at the same time, discouraging microorganisms from colonizing the surface is a very desirable and advantageous feature. High-value materials of this type are gaining increasing attention.

In this review, we have focused on research related to self-healing siloxane materials with antibacterial and antibiofilm properties that were directly designed for or have the potential to be applied in the area of flexible electronics but also for biomedical devices and as antifouling coatings.

## 2. Antimicrobial Properties of Polymeric Materials

A significant interest in antimicrobial polymers has been observed in the recent decade due to their high and long-lasting activity and enhanced passive or active antimicrobial properties [10,11,12,13,14,15]. The application of antimicrobial macromolecular materials can be an excellent way to counter microbial infections, including bacterial cell deposition and biofilm formation. There are several modes of action for antimicrobial polymers (Table 1). Such systems are more environmentally friendly, and good results can be achieved even with relatively low concentrations of biocides combined with polymeric materials. Their enhanced selectivity and lower risk of acquiring resistance to active agents by bacteria should be stressed.

Organosilicon polymers are an important group of macromolecular materials, both for technological and application reasons, as well as for the possibility of imparting biocidal properties [16,17,18]. The type of substituent in polymeric silicone materials determines their antimicrobial activity. The early research on antimicrobial silicones was focused on materials containing ionic QAS groups [19,20,21,22,23,24]. The structure and hydrophobicity of QAS, including the size of the cationic charge and the morphology of the alkyl chain, govern the antimicrobial activity of polysiloxanes [25,26,27]. Although QAS substituents increase polysiloxanes' solubility in water and make their action on plankton cells more effective, the same effect prolongs the exposure of other living organisms to these toxic and harmful combinations. Cross-linked silicone elastomers are a much safer solution and exhibit high biocidal activity without adverse environmental effects [28,29].

Silicone elastomers, to acquire antibacterial properties, can also be doped with metal NPs, such as Ag NPs [30,31,32,33], Au NPs [34,35] as well as copper and zinc oxides [36], and Cu-based metal-organic framework polymers (MOFs) [37]. Organic compounds, well known for their antimicrobial properties (for example, triclosan), were also used to prepare antimicrobial silicone-based composites [38,39]. The antimicrobial and antibiofilm properties of superhydrophobic silicone coatings were derived as a result of the synergistic effects of the active components used (e.g., a composite of MOF, triclosan, epoxy resin, and polydimethylsiloxane [40]). Recently, our group has implemented a novel approach for modification of silicones with specific organic groups, including grafting polysiloxane backbones with naturally occurring bioactive phytochemicals: N-acetylcysteine [41,42], eugenol, and linalool [43] as well as polar but not ionic 2-(carboxymethylthioethyl)-, 2-(n-propylamidomethylthioethyl)-, and 2-(mercaptoethylamidomethylthioethyl)- groups [44]. We have shown that the specific functionalization of polysiloxanes may increase their antiadhesive properties against a range of prokaryotic and eukaryotic microorganisms of different cell organization and cell wall structures, irrespective of the properties (hydrophobicity or hydrophilicity) of silicone materials.

## 3. Self-Healing Polymers—Concise Overview

Self-repairing macromolecular materials have gained tremendous interest due to their properties and potential for use in a wide range of applications. The term “self-healing” refers to their ability to regain structural or molecular integrity after physical damage (scratches, cracks, cuts, or delamination) and molecular breakage. Such features can increase their durability and longevity and enable them to be employed in harsh environments. At the molecular level, the mechanism of self-repair can be chemical (incorporation of dynamic bonds), physical (inter-chain diffusion, formation of separated phase morphology, shape memory effects), or, alternatively, a combination of the two approaches. The detailed chemistry and mechanisms behind self-healing have been extensively reviewed [45,46,47,48,49,50,51]. It must be stressed that successful repair requires sufficient mobility and entanglement of polymer chains, which help reform broken bonds and facilitate the diffusion of healing agents toward the damaged part. The same phenomena are the cause of phase separation in polymeric materials, which can also be used for spontaneous repair. The self-healing of silicone elastomers can be extrinsic or intrinsic (Table 2).

Extrinsic self-healing is typically based on the release of an embedded or encapsulated curing agent to seal micro-fractures in the polymer matrix. This approach is often implemented in thermosets and represents a combination of both physical- and chemical-based self-healing. The durability of S-H effect achieved with the extrinsic approach is restricted by the capacity of micro-vessels carrying the active component. The complexity of designing and manufacturing extrinsic self-healing materials and their high sensitivity to environmental conditions have led to a growing interest in reversible systems that can break and heal repeatedly under external stimuli.

Intrinsic chemical reconstruction of a polymer can be achieved by incorporating dynamic covalent or non-covalent bonds. Dynamic bonds can be reversibly formed or reoriented to reverse deformation or damage. Healing in such systems can be repeated many times. Many of these processes occur spontaneously; however, some are activated or enhanced by heat or light. All the re-mending strategies can be applied to silicone-based materials and elastomers, provided the polysiloxane chains are suitably functionalized.

### Self-Repairing Polysiloxanes—General Healing Mechanisms

Several comprehensive reviews have already been devoted to the topic of self-healing PDMS-based materials [5,6,7,8,9,52]. In general, there are two main types of S-H siloxane elastomers that follow either an extrinsic or intrinsic self-healing route. Extrinsic self-healing of silicones is based on some specific reactions, such as condensation curing, radical reactions, and hydrosilylation, and often involves the use of microvesicles containing reactive components. Early self-healing systems designed for poly(dimethylsiloxane) elastomers operated through the use of microcapsules containing a crosslinking agent that were incorporated into a silicone matrix. The healing agent was released into the cracked interfaces during the healing process. Such materials typically have a short service life as a limited number of healing cycles can be performed due to the exhaustion of the reactive crosslinking agents.

In contrast, the operation lifetime of intrinsic self-healing silicones is much longer since their re-mending is based on various bonding mechanisms that involve the exchange of supramolecular or dynamic covalent bonds between specific structural entities. Mechanical damage in such materials can be fixed by hydrogen bonding, the formation of disulfide bonds or reversible Diels–Adler adducts, metal-ligand coordination, ionic interactions, van der Waals forces, or π-stacking. The healing rate depends on the type of interaction. Some elastomers can be healed under ambient conditions (room temperature and no external stimuli) within a couple of days. Temperature may enhance the process because of the more effective diffusion of polymer chains. Dynamic covalent bonds typically require thermal (DA reactions) or UV-light (disulfide bonds) treatment.

The type of interaction not only governs the effectiveness of the S-H process but also affects the mechanical properties of the crosslinked elastomer. Weaker interactions (e.g., weak hydrogen bonds) make the polymer soft, ductile, and pliable. Strong supramolecular interactions (multiple hydrogen bonds) and dynamic covalent bonds bring in more rigid crosslinks and make the elastomers tougher.

The effectiveness of S-H depends not only on the type of chemical reactions involved in a given system. Both the dynamic nature of the siloxane bonds and the diffusion of a bulk silicone material are equally important for successful self-healing [53]. Each component, especially hard segments inside macromolecules that may prevent diffusion of siloxane chains, can limit local dynamics within the supramolecular network and decrease the efficiency of self-healing. Chain diffusion can be accelerated at higher temperatures due to enhanced chain mobility and the transfer of healing factors toward the damaged area. This problem becomes particularly important when hybrid particles are added to improve the thermal and/or mechanical properties of silicone elastomers. They can provide a diffusion barrier, especially in the case of strong filler/polysiloxane adhesion and agglomeration of the filler grains.

Tuning the strength of such interactions through chemical design, along with adjustment of the length of polysiloxane segments, can be a means to achieve silicone materials of excellent durability (in terms of S-H characteristics) and outstanding mechanical properties. New generations of self-healing siloxane elastomers are typically based on multiple dynamic interactions of different strengths. A variety of available noncovalent supramolecular contacts and dynamic covalent bonds allow for precise design of the properties by adjusting the strong and weak associating groups and tuning the strength of interactions in the phase-separated domains.

## 4. Self-Healing Polysiloxanes of Antimicrobial Properties

Polymeric materials that have good anti-biofouling properties can lose their advantageous functions through physical damage and chemical degradation when even minor scratches occur. Preventing biofouling in such cases can be challenging. That is why the development of durable antifouling polymeric materials and coatings with healing properties is so important. Self-repairable materials, especially those inspired by the healing processes of natural organisms, are a rapidly developing and current research topic. It has been shown that such antimicrobial macromolecular systems can self-repair under suitable conditions [54]. Microbial infections and biofilm formation create a problem that can be particularly important for silicone materials exposed to adverse conditions. New strategies for the development of high-performance antifouling materials may be provided by a proper design of self-healing mechanisms to restore original matrix continuity and mechanical strength. There are several groups of S-H polysiloxane materials with an in-built ability to repel microorganisms (Table 3). Their characteristics are presented in the following subsections.

### 4.1. Self-Healing by Means of Hydrogen Bonding in Antimicrobial Silicones Containing Urea Motifs

**Scheme 1:**
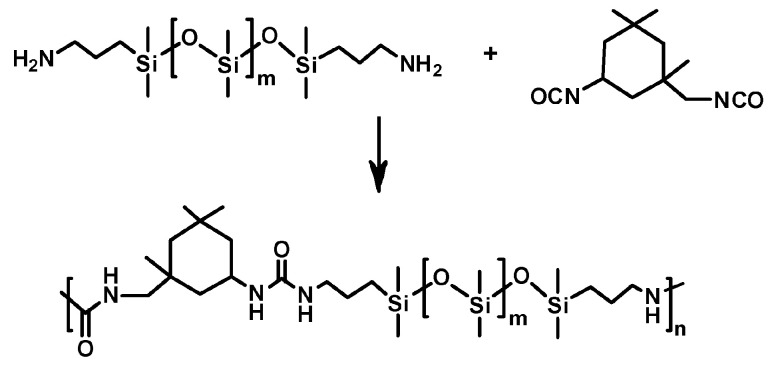
Synthesis of PDMS-PUa.

Urea units in the structure of macromolecules bring in a self-healing potential through the action of hydrogen bonds. A polymer coating that exhibited good adhesion to substrates and S-H characteristics at room temperature in air or artificial seawater was obtained with PDMS-based polyurea (PDMS-PUa) (Scheme 1) and a small amount of organic antifoulant 4,5-dichloro-2-n-octyl-4-isothiazolin-3-one (DCOIT) [55].

The comparison of stress-strain curves recorded for the original and self-repaired PDMS-PUa as a function of time showed that although after 24 h of S-H, the stretchability of PDMS-PUa (elongation of 551%) has recovered, its ultimate strength (0.81 MPa) has not been restored. Both the ultimate strength and the elongation were regained after 48 h of healing. Increasing the temperature accelerated the repair process based on the breaking and reconstruction of hydrogen bonds. The addition of DCOIT influenced the tensile strength and repair efficiency of PDMS-PUa. The ultimate tensile strength varied inversely with the concentration of DCOIT, while both elongation at break and self-healing rate improved with increasing its content. With 5.0 wt% of DCOIT, the ultimate strength of PDMS-PUa was 0.44 MPa, and the self-repairing efficiency reached about 98%. However, when the content of DCOIT reached 10.0 wt%, the tensile strength decreased to 0.22 MPa, despite the complete self-repairing. The S-H effect was based on the formation of hydrogen bonds between the carbonyl group in DCOIT and NH groups in PDMS-PUa, while the reduction in tensile strength could be explained by the weakening of interactions between PDMS-PUa macromolecules, which are distanced from each other by the long alkyl chains of DCOIT molecules. The plasticization of PDMS-PUa by DCOIT enhanced the mobility of polymer chains, which was beneficial for self-healing. PDMS-PUa/DCOIT maintained its self-repair properties in the marine environment (at 5.0 wt.% DCOIT, the strength and elongation of the damaged material were almost restored after 48 h).

**Scheme 2:**
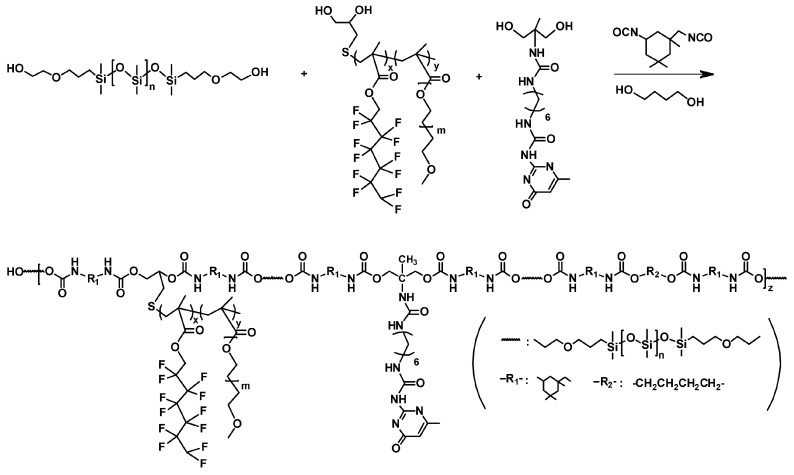
Synthetic route to the self-healing polymer PDMS-UPy-T_x_.

A drawback of this system is that the effectiveness of active agents that are not covalently attached to the polymer matrix may decrease with time. More permanent activity was achieved with specialty polymers, such as a silicone-based polyurethane containing 2-ureido-4[1H]-pyrimidinone (UPy) units and amphiphilic fluorocarbon pendant chains (PDMS-UPy-T_x_; Scheme 2) [56]. The quadruple hydrogen bonding between UPy provided self-healing, whereas the fluorocarbon segments reduced protein adsorption and adhesion of marine bacteria and diatoms.

Both UPy and Ua groups can form hydrogen bonds with various substrates; therefore, the adhesion strength of the coating was significantly improved (0.9–3.0 MPa, depending on the type of substrate) compared with a typical polydimethylsiloxane elastomer (0.3–0.4 MPa) that can adhere only through Van der Waals forces. A decrease in the adhesion strength of PDMS-UPy-T_x_ occurred with increasing the content of amphiphilic telomer T. The increase in surface hydrophilicity at high levels of T-units resulted in swelling of the coating and a decrease in toughness. Despite this effect, the adhesion strength of PDMS-UPy-T_15_ (the highest studied amount of T) was still higher than 1 MPa. The surface energy of the modified polymeric coating and elastic modulus were low (24 mJ m^−2^ and 1.9 MPa, respectively).

Furthermore, the increase in self-healing efficiency was proportional to the telomer content. PDMS-UPy-T_0_ (no amphiphilic F-PEG telomers) had the highest tensile strength (5.34 MPa) and toughness (17.10 MJ m^−3^) but the lowest self-healing efficiency. PDMS-UPy-T_15_ showed full recovery of mechanical strength after healing. This can be explained by the fact that although the incorporation of UPy strengthens the matrix through the formation of crosslinks via quadruple hydrogen bonds, the same effect limits the mobility of polysiloxane chains. Incorporation of the telomers disrupts the hydrogen bonding between polymer chains. The resulting enhancement of mobility of PDMS-UPy-T_x_ was reflected in a decreased glass transition temperature (*T*_g_). This is also in line with the observed improvement in S-H performance at higher temperatures. An increase in the telomer content lowered the tensile strength but improved the elasticity of PDMS-UPy-T_x_ (stretchability up to 1000% of its original length for PDMS-UPy-T_15_).

**Scheme 3:**
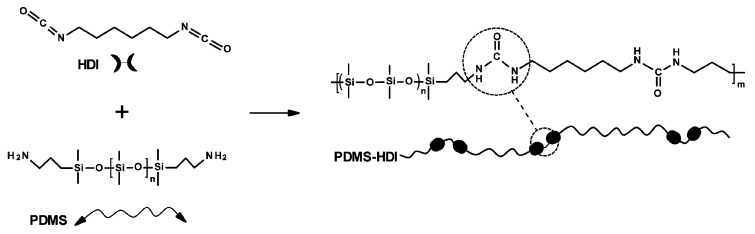
Synthesis of isocyanate-modified PDMS.

Despite the very interesting results obtained with PDMS-UPy-T_x_, environmentally-oriented research should rather be focused on fluorine-free solutions. This requirement was fulfilled by an isocyanate-modified PDMS (I-PDMS) coating with mechanical durability, self-healing ability, and low surface energy with anti-corrosion/biofouling potential [57]. The polymer chains were physically crosslinked by dynamic intermolecular hydrogen bonds between the urea groups (Scheme 3).

**Scheme 4:**
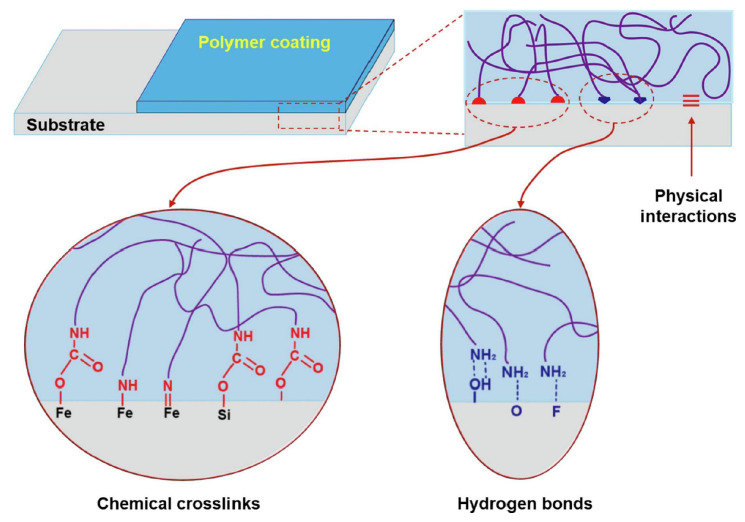
Interactions between I-PDMS and substrates of various types. Reproduced from Ref. [57] with permission from Elsevier.

The low surface energy of the I-PDMS coating was adjusted by changing the length of the polydimethylsiloxane segment (i.e., ~13.1 mJ/m^2^ and ~28.8 mJ/m^2^, respectively, for polysiloxane of Mw ~30,000 and ~3000). The effect was attributed to changes in the mobility of polymer chains and the density of the organic crosslinks. The hydrophobicity of the I-PDMS coating was retained after 500 cycles of adhesive tape-peeling or 200 cycles of sandpaper abrasion. It was also shown that I-PDMS can effectively reduce corrosion and biofouling on metal substrates, implying its promising practical potential as a protective coating. The observed results of different adhesions of I-PDMS films to various substrates were explained in terms of different interfacial interactions (Scheme 4).

**Figure 1:**
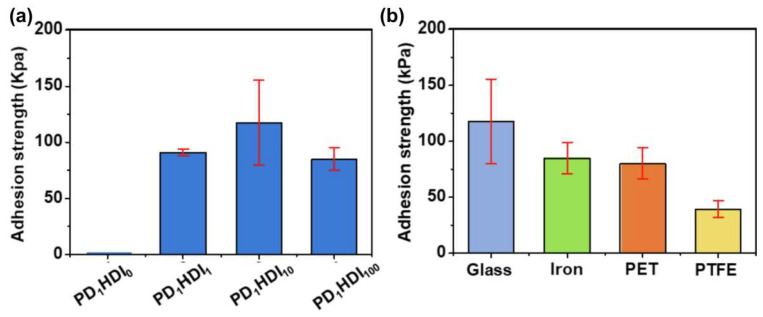
Adhesion strength of the I-PDMS coatings: (**a**) with different molar ratios of PDMS to HDI on the glass substrate; (**b**) of PD_1_HDI_10_ coatings on different substrates. Reproduced from Ref. [57] with permission from Elsevier.

Chemical and chelation bonding that involved metal atoms and residual isocyanate, amino acid, or other surface-reactive groups (e.g., Si–COO–NH, Fe–COO–NH, Fe=N, Fe–NH), dominated the adhesion in the case of hydrophilic substrates (glass and iron). Additionally, physical interactions, including hydrogen bonds and van der Waals interactions, improved the strength of interfacial adhesion. On the other hand, interactions between I-PDMS and the nonpolar PTFE substrate were only physical. Although they resulted in small adhesion strengths (Figure 1), they were still superior to those reported for most coatings on PTFE. The effect was attributed to the large electronegativity difference between fluorine atoms in PTFE and hydrogen atoms in hybrid I-PDMS. It could induce dipole-dipole interactions, which extremely enhance van der Waals contacts, and would allow for wetting of the PTFE surface by I-PDMS.

### 4.2. Self-Healing of Antimicrobial Silicone Elastomers Admixed with Natural Compounds as Additives/Modificators

**Scheme 5:**
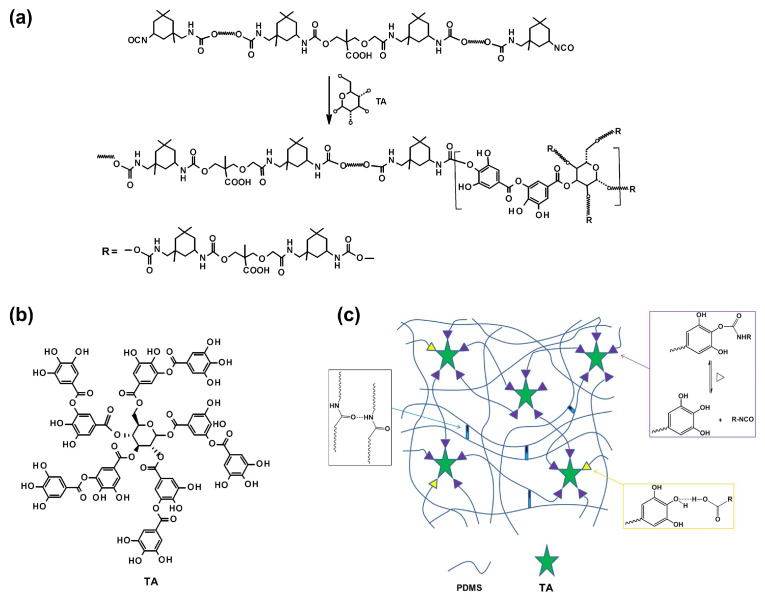
Synthesis of the PDMS_1_-TA_x_ elastomers (**a**); chemical structure of tannic acid (**b**); and the structure of a dynamic network based on PDMS_1_-TA_x_ (**c**). Adapted from Ref. [58] with permission from John Wiley and Sons.

Silicone elastomers with improved antibacterial, flame retardant, self-healing, and recyclable functions were obtained using tannic acid (TA) as the cross-links precursor, polydimethylsiloxane as a soft chain segment, and 2,2-bis(hydroxymethyl)propionic acid as an intermediate chain extender (Scheme 5) [58]. Tannic acid is a naturally antimicrobial substance; thus, >90% antimicrobial efficiency against *E. coli* and *S. aureus* was observed, and a final oxygen index of 25.5% was obtained in this system. The composition became increasingly effective with the increase in the TA fraction.

The unique structure of the tannic acid with multiple phenolic groups also helped to improve the mechanical properties of the elastomer. The performance of the hybrid composites was restored after the self-healing. The S-H mechanism originated from the phenol-carbamate bonding and hydrogen bonding interactions. PDMS_1_-TA_x_ combined both good mechanical and tensile properties, with high tensile stress and high elongation at break. At higher molar ratios of TA to PDMS, the elongation at break decreased; however, the tensile stress gradually increased. The best overall performance was achieved for the sample with a tensile strength of 7.5 MPa and 1540% elongation at break.

Dynamic mechanical tests at different frequencies showed that the storage modulus (*E*″) gradually increased with frequency increase, while the loss modulus (*E*′) remained constant. Cyclic tensile tests that were used to evaluate the dissipation energy of PDMS_1_-TA_2_ showed a significant hysteresis in the first load-unload cycle, indicating high energy dissipation through hydrogen bond breakage. The effect was significantly reduced in the subsequent cycles, attributable to insufficient reformation of hydrogen bonds within the experiment time scale.

PDMS_1_-TA_x_ elastomers exhibited excellent self-healing due to the dual action of dynamic hydrogen and carbamate bonds. The S-H increased with time, and the healing efficiency in the case of PDMS_1_-TA_2_ reached more than 85% after 18 h at 85 °C. In addition, the presence of TA may be of importance regarding the flame retardancy of the hybrid material, as TA chars when exposed to flame.

Another interesting example is a PDMS-based poly(urea-thiourea)/tannic acid composite (PDMS-P(Ua-TUa)-TA) with antifouling properties and efficient self-healing [59]. Excellent mechanical properties (ultimate strength: 2.47 MPa; stretchability ~1000%) were achieved owing to the presence of thiourea and urea groups of different proton donor capacities. A proper balance between dynamic healing and material strength could be designed. Interactions between well-dispersed tannic acid molecules and urea or thiourea groups resulted in multiple cross-linking sites before the migration of siloxane chains. Scratches on the film disappeared completely within 12 min, the repair efficiency of mechanical strength reached 98.4% within 3 h under ambient conditions, whereas the healing efficiency in artificial seawater was 95.1%.

**Scheme 6:**
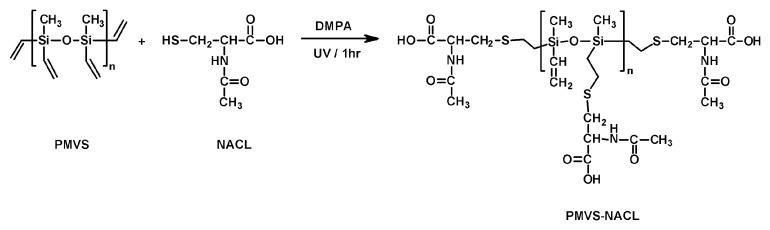
Grafting of NACL onto polysiloxane macromolecules.

Another hybrid self-healing silicone (PMVS-NACL) with antimicrobial properties was obtained by grafting side N-acetyl-L-cysteine (NACL) groups via a thiol-ene addition to polysiloxane with vinyl groups (Scheme 6) [60]. NACL is a biobased amino acid known for its mucolytic properties and PMVS-NACL of different content of the active NACL residues were studied for their potential as antimicrobial coatings.

Macromolecules of PMVS-NACL can form a crosslinked network through strong hydrogen bonds involving carboxyl and acetyl groups. The tensile strength of PMVS-NACL depended on the content of NACL (tensile strength of 4.36 MPa and 586% strain were noted for PMVS-30%NACL). The number of dynamic crosslinks increased with the increase in NACL amount, and optimal results were obtained with 30% vinyl group grafting. The larger number of crosslinks in PMVS-40% NACL made the material rigid and brittle. The S-H effectiveness also depended on the number of NACL, and ~97% was achieved for PMVS-30%NACL. Slightly poorer results were obtained with lower NACL contents. Larger amounts of NACL enhanced the inhibitory effect of PMVS-NACL on *S. aureus*. Yet, it should be stressed that the effect was obtained for polymers of high molecular weight (Mw = 330 kg mol^−1^). Shorter polysiloxanes grafted with NACL, despite their antibiofilm action against a range of bacteria, were found to be soluble in water [41,42] and thus the crosslinking with NACL cannot be regarded as stable.

An enhancement of the mechanical properties of coatings prepared with PMVS-NACL was observed when a part of the carboxyl groups of NACL was neutralized with sodium ethoxide, resulting in the formation of ionic aggregates [61]. Lap shear and peel strengths, with maximum values of 8.16 MPa and 20.48 N/m, were noted at ambient temperature for coatings on metallic Fe, 3.56 MPa and 14.99 N/m for metallic Al, and 5.55 MPa and 20.52 N/m for metallic Cu, respectively. PMVS-NACL, modified in this way, still inhibited the growth of S. *aureus*.

### 4.3. Self-Healing of Antimicrobial Silicone Coatings by Interactions with Zn^2+^ Ions

**Scheme 7:**
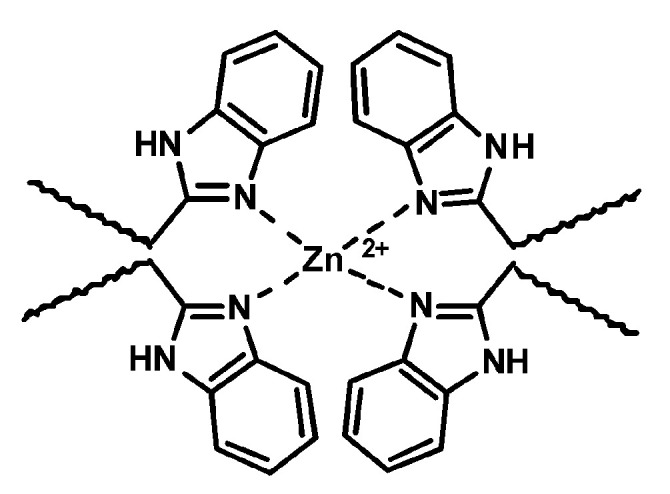
Coordination between 2-benzimidazolyl groups and zinc ions.

Metal oxides can participate in non-covalent interactions based on coulombic attraction between oppositely charged ions that provide high binding strength. In self-assembling systems, the effect of such ionic interactions can be enhanced by the joint action of a large number of active bonds. In such cases, ionic clusters involving metal oxides limit the mobility of polymer chains. Consequently, it provides the polymer matrix with a high modulus but adversely affects chain diffusion and self-repair efficiency. Nevertheless, thermally reversible ionic bonds can bring in recyclability and shape memory for polymers. For example, ZnCl_2_ was a component of a metallo-supramolecular swellable network of poly(N-(2(pyridin-4-yl)ethyl)acrylamide-Zn(II))-l-polydimethylsiloxane (PNP4EA-Zn(II)-l-PDMS) with scratch healing ability [69]. The co-network was attached to the surface of a glass support as a coating that could swell in organic solvents while maintaining dimensional stability. Zn^2+^ ions were involved in the formation of complexes that served as reversible supramolecular cross-links. The scratch-healing polymer coating designed in this way was resistant to delamination. The fact that the polymer material is attached to the surface by coordination bonds rather than covalent bonds prevented the coating from peeling off and allowed for effective self-healing through a multidimensional change in the polymer volume. PDMS coatings with low surface energy and improved adhesion were reversibly cross-linked via coordination bonds between 2-(2-benzimidazolyl)ethanethiol (BET) groups and Zn^2+^ ions [62]. The BET-modified PDMS retained low surface energy and exhibited improved adhesion strength compared to native PDMS due to the synergistic effect of dynamic metal-imidazole (BET/Zn^2+^) coordination complexes (Scheme 7) and hydrogen bonding. Supramolecular interactions are responsible for the reversible cross-linking, and thus the coating exhibited good self-healing in air and in artificial seawater at room temperature. The silicone material containing non-leaching Zn^2+^ ions also exhibited significant fouling resistance against the marine bacteria *Pseudomonas* sp. and the diatom *Navicula incerta*.

**Scheme 8:**
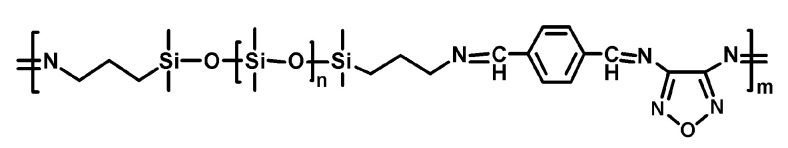
Chemical structure of PDMS-TPA-DAF.

Another silicone elastomer (PDMS-TPA-DAF-Zn) (Scheme 8) gained antibacterial activity and high self-healing ability in the coordination of Zn^2+^ ions with imine bonds [63]. The polymer was prepared with α,ω-aminopropyl-terminated polydimethylsiloxane (PDMS, Mn = 3 kg mol^−1^) in a Schiff base reaction with terephthalaldehyd (TPA) and 3,4-diaminofurazan (DAF), followed by treatment with ZnCl_2_.

**Figure 2:**
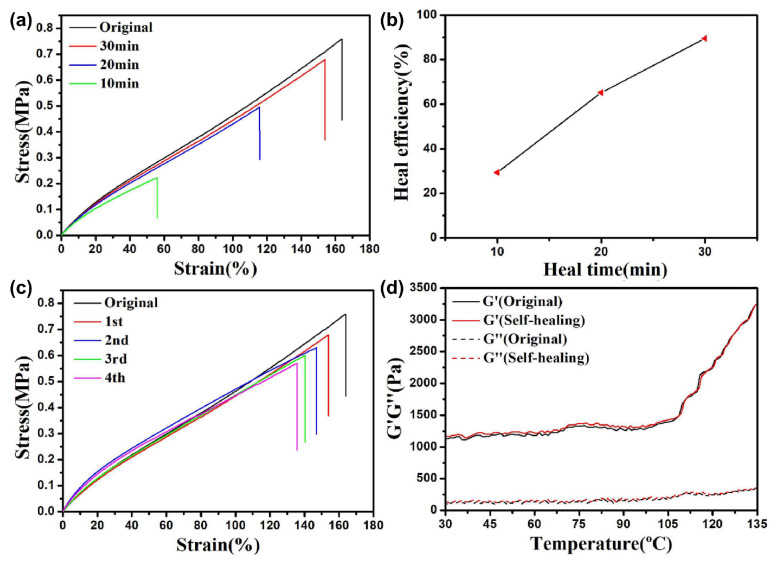
Mechanical properties of PDMS-TPA-DAF-Zn with the Zn^2+^/DAF molar ratio of 1:8: (**a**) tensile curves before and after self-healing for different times; (**b**) heal efficiency after curing for different times; (**c**) stress-strain curves with cycle repair four times; (**d**) time sweeps of the elastomer before and after the self-healing. Reproduced from Ref. [62] with permission from Elsevier.

The presence of Zn^2+^ ions and the 1,2,5-oxadiazole heterocyclic structure of DAF are synergistically responsible for the antibacterial action of PDMS-TPA-DAF-Zn. The antibacterial efficiency of this material against *E. coli* and *S. aureus* was very high (respectively, 99.9993% and 99.9997%). The elastomer displayed high self-healing efficiency after thermal treatment at 100 °C and good mechanical properties (Figure 2). The first S-H efficiency was 89.5%. After repeated repairs, the mechanical properties were not significantly reduced, which suggests an efficient recovery of coordination bonds.

ZnO particles were also shown to affect the S-H properties of other hybrid silicone polymers. For example, polydimethylsiloxane (PDMS)-polythiourethane (PTU) composites modified with tetrapodal-shaped micro-nano zinc oxide particles (t-ZnO) were designed as mechanically durable marine fouling-release coatings [70]. The surface energy, mechanical properties, and fouling-release performance evaluation showed that, among all the studied variations (1–5 wt.% of t-ZnO), PTU/1 wt.% PDMS composites with 1 wt.% t-ZnO particles had superior properties and are suitable for applications as marine fouling-release coatings. The influence of t-ZnO (1 wt.%) on the mechanical properties of composites was remarkable. The mean tensile strength was increased by ~20%, while the elongation at break was enhanced by ~30%. The composites with filler amounts higher than 1 wt.% showed similar mechanical properties. The results corroborate the earlier reports on enhanced tensile properties at ≤5 wt.%. A further increase in ZnO content deteriorated the mechanical properties of the polymer composites due to particle agglomeration and stress concentration [71]. It was supported by the variations in t-ZnO domain size and their distribution in PDMS, as evidenced by confocal laser microscopy and EDX measurements. PTU/PDMS samples contained silicone microdomains of 50–200 µm size. Although homogeneously distributed, they were detectable even at only 1 wt.% t-ZnO.

It was also shown that mechanically strong, highly stretchable, and self-healing thermoplastic silicone elastomers (TPSE) can be formed through a simple physical blending of polysiloxanes grafted with carboxylic groups (PDMS-*g*-COOH) and ZnO [64]. The cross-linking was attributed to coulombic interactions. FT-IR spectroscopy confirmed the participation of COOH groups in the formation of PDMS-*g*-COOH/ZnO composites. Unfortunately, no information on the antibacterial properties of these composites was provided.

The mechanical properties (tensile strength and elongation) as well as the self-healing abilities of the dynamic ionically bonded networks were modulated by varying the molecular weight of PDMS precursors and the molar ratio of COOH/ZnO. The results obtained for PDMS-*g*-COOH/ZnO with dynamic mechanical analysis, tensile, and rheological measurements proved an excellent mechanical strength of (>5 MPa) and good elasticity (up to 1300% of the original PDMS-*g*-COOH/ZnO sample length of 16.1% molar percentage of COOH groups and Mw = 107.3 kg mol^−1^). Efficient self-healing recovery (83.5% at 80 °C for 4 h) was also demonstrated. Adjusting the number of coordination sites also affected the mechanical properties of PDMS-g-COOH/ZnO composites. A completely cross-linked network should be formed at the molar ratio of COOH/ZnO = 2. DMA traces exhibited two *T*_g_s of −84 °C and 57 °C, indicating the glass transition of the PDMS main chain and the disassociation of ionic bonds, respectively. Too high or too low a molar ratio of COOH/ZnO could adversely affect the properties of the dynamic network. In the former case, incomplete salt-bonded clusters were generated (resulting in low strength but good ductility of the material), whereas, for lower amounts of COOH groups, aggregation of ion pairs and formation of local microdomains may occur, leading to an enhancement of the elastic network but a decrease in ductility.

Nanoparticles of ZnO were also loaded on the surface of multiwalled carbon nanotubes, and the hybrids were used as microwave absorbers in a silicone-based composite (MWCNTs)/DA-PDMS self-healable via Diels-Alder chemistry (no information on antibacterial properties) [72]. A superhydrophobic and self-healing coating containing ZnO-encapsulated mesoporous polydopamine (m-PDA) microspheres exhibited very good antibiofouling ability and antibacterial action against *E. coli* and *S. aureus* [65]. ZnO-NP encapsulated the microspheres via electrostatic interactions and hydrophobic interactions with PDA and amino-modified silicone oil (ASO) that was loaded in the microspheres. The hybrid particles were UV/NIR/acid/base stimulus-responsive. ZnO-NP acted simultaneously as the protective layer for the m-PDA and functional filler in the coating, which contributed to the release of ASO and the antibacterial action of the coating.

### 4.4. Self-Healing of Gallium-Based Antimicrobial Silicone Coatings

Gallium-based liquid metal (GLM) is non-toxic and exhibits good antibacterial properties, which makes it suitable for antifouling applications. Gallium ions (Ga^3+^) play a major role in this process as they can substitute for Fe^3+^ ions. However, the inability of Ga^3+^ to undergo one-electron reduction disrupts bacterial iron metabolism, leading to the death of microorganisms [73]. Ga^3+^ ions can also generate reactive oxygen species (ROS) that cause mutations and the death of bacterial cells. Moreover, GLM nanodroplets can change their shape in a low-intensity rotating magnetic field [74]. The sharp edges of microparticles formed in this way can rupture bacterial biofilm.

GLM nanodroplets, functionalized with a zwitterionic polymer (polyethylenimine-quaternized derivative, PEIS), were incorporated into a PDMS coating as a functional filler [66]. The well-dispersed PEIS-GLM nanodroplets displayed very good anti-bacterial properties and antifouling performance due to the synergistic activity of Ga^3+^ ions and the quaternary ammonium groups in PEIS. The removal of >90% bacteria (*S. aureus* and *E. coli*) and 70% microalgae (*Dunaliella* and *Porphyridium*) was evidenced. It was also shown that PEIS-GLM endows the PDMS coating (Sylgard 184 silicone-elastomer base and curing agent) with self-healing properties under external mechanical force. The phenomenon was explained by crosslinking based on free radical polymerization of residual vinyl groups in Sylgard 184, initiated by partially exposed gallium atoms [75]. As a result, the damaged coating was repaired, and the corrosion of the substrate was hindered.

Similar results were obtained on the replacement of PEI with a zwitterionic dopamine sulfonate ligand (ZDS) [67]. The ZDS@GLM nanodroplets presented antibacterial and antifouling properties (>50% of *S. aureus* and *E. coli* and 65% of *Porphyridium* were removed). Slightly poorer antimicrobial action can be linked to a lower number of ionic species in ZDS than in PEI. Analogously to PEI-GLM, ZDS@GLM endows the PDMS coating with self-healing properties. The surface of GLM was also modified with tannic acid (TA), grafted with polyethyleneimine (PEI) via Schiff-base reaction, and/or Michael addition to derive poly(TA/PEI)-functionalized GLM nanodroplets (GLM-PEI) that were subsequently modified with triisopropylsilyl methacrylate (TISM) [68]. TISM reacted with the amine group of poly(TA/PEI) through aza-Michael addition to form zwitterionic structures. Zwitterionic GLM-TISM were then dispersed in PDMS elastomer (Sylgard 184) to form nano-composite coatings (PDMS@GLM-TISM). The coating displayed very good antifouling action (inhibition of >96% of bacteria, *E. coli* and *S. aureus*, and 77% of algae), which was attributed to the synergy of bactericidal GLM nanodroplets and strong surface hydration caused by zwitterionic groups. The functional nanodroplets were also responsible for the observed self-healing effect by crosslinking residual vinyl groups in the Sylgard matrix.

## 5. Applications of Self-Reparable Antimicrobial Polysiloxanes

Research interest in developing self-reparable, fouling-release, and corrosion-resistant polymer coatings has been growing during the last decade (Table 4) [6,76]. Enhancement of adhesion strength may adversely affect antifouling properties. Low wear resistance also reduces the anticorrosion effect. That is why the focus on the antifouling action of silicone coatings is accompanied by efforts to make them resistant to mechanical damage. Self-healing is a very attractive option, yet a special design of the chemical structure is required to support the action of environmental factors, e.g., seawater.

### 5.1. Antifouling Coatings

Polydimethylsiloxane-based polymers have been extensively studied for the development of fouling release coatings. Hydrophobicity and low elastic modulus make it difficult for marine organisms to attach to the surface of silicone films. Yet, the application of such coatings to polar substrates, including metals, is not facile, and thus their longevity is poor. Several research papers were devoted to silicone materials of this type, mainly designed for action against undesired colonization of bacteria, microalgae, and molluscs that can adversely affect ships and marine equipment. Toxic antifouling paints containing tributyl tin (TBT) based compounds have been banned, and there is a need for antifouling coatings that are innovative and efficient but safe for the environment.

Self-healing antifouling silicones have been prepared by the insertion of thiourea and ether groups into a silicone resin [77]. A polyether-thiourea-siloxane (PTS) copolymer of narrow molecular weight distribution was synthesized via free radical polymerization. Thiourea residues in PTS formed an H-bonded network, and it was used as an additive to antifouling coatings based on crosslinked PDMS in a mixture with phenylmethylsilicone oil (PSO). Incorporation of a small amount of PTS/PSO was enough to increase surface micro-roughness and hydrophobicity as well as improve the mechanical properties of the coating, including elongation at break. With an increasing amount of PTS, phase separation was promoted, and PSO encapsulated in the copolymer exhibited enhanced leaching onto the coating surface. The H-bonding between thiourea groups improved the adhesion strength between the coating and the substrate only up to a certain limit. The most effective antifouling performance was achieved with a composition of 12 g of PTS in 100 g of the PDMS network. Excessive amounts of PTS reduced the crosslinking density and deteriorated the mechanical performance of the coating.

A self-healing antifouling coating of excellent mechanical strength was prepared with the amphiphilic block copolymer polydimethylsiloxane-poly(2-(dimethylamino)ethyl methacrylate) (PDMS-PDMAEMA) that was introduced into the polydimethylsiloxane-based polyurethane (PDMS-PU) matrix and subsequently zwitterionized during solidification [78]. Polysiloxane segments of both polymers entangled to form a semi-interpenetrating network while the zwitterionic units migrated toward the external parts on immersion in water. This effect provided the hybrid coating with antifouling properties, especially the resistance towards adhesion of proteins (156 μg/cm^2^ vs. 14 μg/cm^2^) and marine microorganisms (425 × 10^3^ n/cm^2^ vs. 12 × 10^3^ n/cm^2^). Swelling of the zwitterionic segments increased the contact fusion rate of the mechanically damaged parts and promoted self-healing of the micro-phase-separated system.

A silicone-based coating with antifouling and anti-corrosion properties self-healed through supramolecular interactions involving hydrogen bonds and dynamic disulfide bond exchange between the functionalized monomers lipoic acid-benzothiazole (LA-BTZ) and lipoic acid-modified PDMS-based polyurea-urethane (PDMS-PUU-LA) [87]. Small scratches on the film disappeared completely within 40 minutes at room temperature in the air. The original toughness (2.58 MPa of ultimate strength) and stretchability (1014.7%) were recovered under ambient conditions or artificial seawater with healing efficiencies of 98.5% (4 h) and 94.6% (8 h), respectively. The adhesion of silicon-based coating epoxy resin and steel was very strong (~2.50 MPa and ~3.33 MPa), and long-term static fouling-resistant properties and good corrosion resistance in salt spray tests were observed.

A sea slug-inspired PDMS-based smart marine antifouling polyurethane coating with UV-responsive and controllable coumarin release was reported [79]. PU was modified by the insertion of coumarin and eugenol. Coumarin groups can undergo reversible dimerization on irradiation with 365 nm UV (release after treatment with 254 nm UV). Free or chain-grafted coumarin molecules can take part in this process, which leads to an efficient S-H of the resin on UV irradiation. The coating was transparent, highly adhesive (>1 Mpa), mechanically robust (Young’s modulus <4.5 MPa), and exhibited low surface energy (20–30 mJ·m^−2^) as well as good antifouling, anti-algae resistance, and antibacterial properties.

Self-healing, antibacterial, and antifouling coatings were also obtained by surface modification with a silicon oxide layer, followed by grafting of PDMS chains as a “lubricant liquid” [88]. As another example, a vinyl-terminated polydimethylsiloxane (Vi-PDMS) was grafted onto various substrates coated with sulfhydryl-modified hollow mesoporous silica (SHHMS) [89]. A lubricant-grafted slippery surface formed in this way was very stable, exhibited good antifouling properties, and effectively decreased the absorption of organic liquids, polysaccharides, and proteins. Functional antifouling and antibacterial materials of good mechanical and self-healing properties were obtained by UV/moisture dual curing of PDMS containing methacryloyloxy and methoxy silane groups (MAPDMS)-microcapsule-SiO_2_ (MPMS) of hierarchical structure [90].

Self-healing and antifouling hybrid materials can also be prepared with the use of nanomaterials as well as artificial biocides or bioactive natural compounds. For example, silver nanoparticles (AgNPs) and polymethyltrimethoxysilane (PMTMS) were introduced into AZ31 Mg alloys via layer-by-layer (LbL) assembly and siloxane self-condensation reaction to obtain an antibacterial coating against *S. aureus* [80]. PDMS was also used as a hydrophobic binder for coating octadecylamine (ODA)-modified flower-like Ni(OH)_2_ particles (Ni(OH)_2_@ODA) that were applied for the modification of cotton fabric [91]. Excellent antifouling and self-cleaning performances, as well as durable superhydrophobicity, were achieved in this system.

4,5-dichloro-2-n-octyl-4-isothiazolin-3-one concentration-dependent antibiofouling activity of PDMS-PUa/DCOIT, based on the release of the antifoulant (1.5–4.5 μg cm^−2^ d^−1^ for 1.0–10 wt% of DCOIT, respectively) was studied [55]. The self-stratifying properties of PDMS-PUa (the urea groups interact more strongly with the surface, while PDMS chains tend to migrate to the upper layer) were altered upon the addition of DCOIT. DCOIT affected the adhesion of PDMS-PUa to the substrate because of its competitive interactions with Ua units. Polydimethylsiloxane polymer containing imine and urea groups (PDMS-UIa) was used as an eco-friendly antifoulant after the addition of a small amount of N-octyl-2-hydroxybenzamide [81].

The bio-adhesive and good bacteriostatic effects of tannic acid improved both the adhesion strength and the antifouling properties of a PDMS-based coating. The macromolecular polyphenol is active against Gram-positive and Gram-negative bacteria, whereas the catechol/pyrogallol groups of TA can interact strongly with substrates through hydrogen bonds, ionic bonds, or hydrophobic interactions. The latter plays a major role in underwater adhesion. Correspondingly, PDMS-P(Ua-TUa)-TA displayed strong adhesion to the substrate (~2.2 MPa) as well as good antibacterial and anti-diatom properties (~96%, ~95%, ~93%, ~84% reduction, respectively, for *Pseudomonas* sp., *E. coli*, *S. aureus*, and diatoms) [59].

### 5.2. Wearable Electronics and Biomedical Devices

**Scheme 9:**
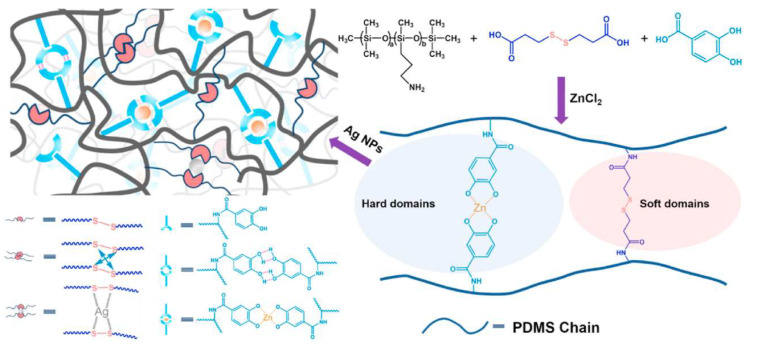
Synthesis and synergistic interactions operate in the SS-PDMS-DH-Ag conductive elastomer. Reproduced from Ref. [86] with permission from Elsevier.

Flexible electronics applications require mechanically durable materials with the ability to accurately monitor electrical signals. A mussel-inspired self-healing antibacterial, conductive organosiloxane elastomer with good mechanical properties was thus designed for wearable strain sensors [86]. The PDMS-based copolymer with side 3-aminopropyl groups was transformed into a supramolecular network (SS-PDMS-DH) by dehydration coupling with carboxyl groups of DHBA, which resulted in the formation of amide bonds. It was further crosslinked by the presence of Zn^2+^ ions and silver nanoparticles (Ag NPs) (Scheme 9).

Tensile properties in this system were controlled by phase separation and multiple reversible interactions. Excellent mechanical properties were achieved by the synergistic effect of Zn^2+^ coordination on DHBA residues and dynamic disulfide crosslinks. The former were responsible for hard segment formation that stiffened the network, while the latter acted as soft domains that contributed to energy dissipation. The addition of Ag NPs to form S–Ag interactions with disulfide bonds reinforced the network and made the elastomer conductive. High tensile strength (2.4 MPa stress) and stretchability (1762% strain) were achieved at 10 wt% nanofiller content. The combination of the bactericidal effect of Ag NPs and zinc ions with the bacteriostatic effect of the hydrophobic surface of PDMS brought in an efficient antibacterial performance of the conductive elastomer against *E. coli* and *S. aureus* (antibacterial efficiency of 98.79% and 98.52%, respectively). The effect was related to the denaturation of bacterial respiratory enzymes by Ag NPs that penetrated cells and the adsorption of Zn^2+^ on cell membranes under the action of Coulomb forces.

**Figure 3:**
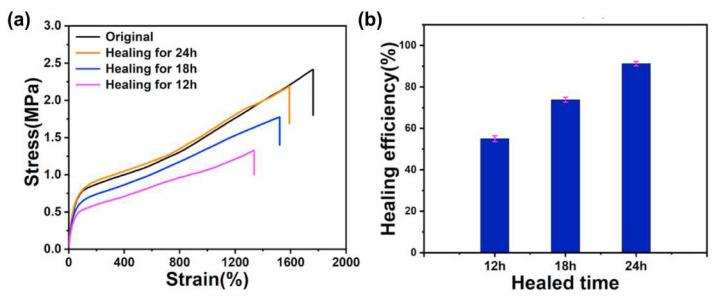
(**a**) Tensile curves of SS_0.4_-PDMS-DH_0.6_-Ag_10_ after different self-healing times at room temperature; (**b**) Self-healing efficiency of SS_0.4_-PDMS-DH_0.6_-Ag_10_ at different healing times. Reproduced from Ref. [86] with permission from Elsevier.

The synergistic effect of multiple dynamic interactions and reversible covalent bonds made the SS-PDMS-DH-Ag elastomer self-repairable. It restored 91.2% of the original mechanical properties after 24 h of spontaneous self-healing at room temperature (Figure 3). In addition, elastomers of the SS_0.4_-PDMS-DH_0.6_-Ag_10_ composition were applied to self-repairing wearable strain sensors for monitoring the bending motions of human joints.

**Scheme 10:**
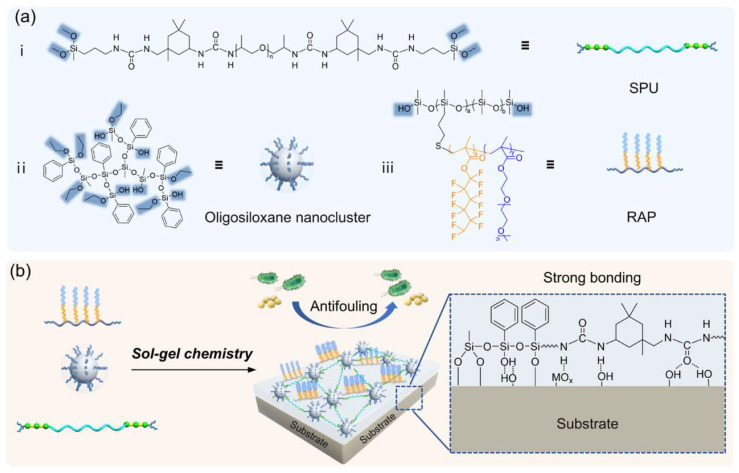
Chemical structure of the components (i, ii, iii) used for the preparation of the SPU/RAP hybrid coating (**a**) and schematic illustration of interactions between the coating and a substrate (**b**). Reprinted with permission from [82]. Copyright (2023) American Chemical Society.

Silanol-terminated PDMS side grafted with fluorocarbon and poly(ethylene glycol) chains was used as a reactive amphiphilic polymer (RAP) in a blend with bis(dimethoxy)methylsilane-terminated polyurea (SPU) and oligosiloxane nanoclusters to form a surface-enriched antifouling coating that displayed superior antimicrobial action and strong substrate adhesion (Scheme 10) [82].

The oligosiloxane nanoclusters with free silanol groups can cross-link the alkoxysilane-terminated flexible SPU by sol-gel chemistry. A tough polymer network was formed and interlinked with the substrate via silanol groups. The low-surface-energy RAP siloxane self-enriches on the surface of the coating. The thin film of this composition is transparent (>85% transmittance), tough (tensile strength of ~12 MPa), and exhibits fouling resistance against proteins and bacteria. It strongly adheres to various substrates, including glass, ceramic, steel, Ti, and epoxy (3−15 MPa), and can possibly be used as an antifouling coating in flexible electronics and medical devices.

Moreover, PDMS-based optical fibers were integrated into carboxymethyl chitosan (CMCS)-protocatechualdehyde (PA)@Fe hydrogels (CMCS-PA@Fe) to obtain hybrid, pH-sensitive devices for real-time monitoring of the wound healing process [83]. The hydrogel operated by chelating PA@Fe with CMCS. A high antibacterial effect (>99.9%) was noted with the help of the NIR-assisted photothermal effect. The pH-sensitive optical fiber/CMCS-PA@Fe hydrogels can adapt to the movement, and wound bacterial infection can be quickly diagnosed by changes in the environment's acidity.

A hyperbranched polysiloxane terminated by multi-amine (HPSi) was reacted with a linear polyacrylate resin (LP) containing pendent sulfobetaine and acetoacetyloxy residues to form dynamic vinylogous urethane groups. The resulting hybrid material (LP-HP) can be used as a multi-functional coating with high mechanical strength, reversible self-healing, and high antibacterial ability against *E. coli* and *S. aureus* (>95%) provided by nontoxic sulfobetaine groups [84]. The structure of HPSi (large number of amine groups and easy segmental motion owing to fewer chain entanglements) and its concentration in LP resin proved to play a key role in adjusting these properties. As the amount of HPSi increased, the LP-HP coating exhibited higher tensile strength and Young’s modulus but lower elongation at the break because of the increased crosslinking density. The optimal parameters (tensile strength of ~17.89 MPa, the toughness of ~5.72 MPa, self-healing efficiency > 92% at 60 °C for 24 h) were obtained for a polyacrylate coating containing 6.3 wt% of HPSi (LP-HP6). The healed sample exhibited high self-healing efficiency (94.07% of the initial tensile strength, 95.81% of elongation at break, and 92.65% of toughness). The mechanical properties of LP-HP coatings were tuned by changing the amount of HPSi to meet different application requirements. Interestingly, a strain-softening behavior, where strain grows but stress reduces, appeared in the stress-strain curve of LP-HP9 after the yield point.

A smart self-healable PDMS-based hydrogel that inhibited protein adhesion and displayed antimicrobial action against Gram-positive (*S. aureus*) and Gram-negative (*E. coli*) bacteria was prepared with curcumin-loaded zwitterionic polymersomes consisting of PDMS and a tri-block copolymer poly([dimethyl-[3-(2-methyl-acryloylamino)-propyl]-(3-sulfopropyl)ammonium)](poly(sulfobetaine)). The polymersomes were mixed with amine-modified PDMS-based polymersomes, then the dual system was crosslinked with an aldehyde-modified PEG via a Schiff-base reaction [85]. The hydrogel displayed self-healing behavior in saline solution due to ionic interlocking between the poly(zwitterionic) segments. This system significantly enhances the bioavailability of curcumin. Sustained delivery of the hydrophobic drug was observed over 72 h. Moreover, the wettable, self-healing, fouling-resistant, transparent, and soft hybrid material efficiently reduced the deposition of a protein (BSA), and thus it can be potentially used in therapeutic contact lens applications.

## 6. Conclusions

Recent years have seen significant advances in the field of self-healing silicone elastomers with antimicrobial and antifouling properties. This is due to the emergence of new application areas for silicone-based materials, such as stretchable electronics, wearable stress sensors, smart coatings, and soft robotics. However, such high-value silicones have much broader interest in many other areas, including marine antifouling coatings, anti-icing coatings, microfluidics, medical devices, electrochemical biosensors, and beyond.

In this manuscript, we have summarized research work on the synthesis, physical and mechanical properties, and applications of self-healing hybrid materials containing polysiloxane segments. Extrinsic and intrinsic self-healing methods are reviewed, which have recently resulted in significant advances in siloxane-based polymer composites and coatings, including multicomponent systems.

This particular branch of silicone elastomer chemistry and materials engineering can be expected to flourish in the near future due to the unique potential that such materials have. Similarly to the trends observed for S-H materials in general, the new generations of self-healing silicones are not based on single mechanisms that lead to the self-repair of the polymer network. The design of their overall characteristics relies on a multi-level combination of several types of dynamic bonds. Antimicrobial properties are highly desirable, and this feature should ideally be incorporated into the self-regeneration mechanism.

The first idea for the use of S-H silicones was antibacterial coatings. Since then, this branch of polysiloxane-based elastomers has developed greatly. Antifouling S-H silicone coatings, especially those for marine applications, are bound to self-heal under special conditions. Nowadays, it is not only about antifouling and self-repair coatings; however, S-H mechanisms are also involved in promoting adhesion to various substrates, including metals, which is not trivial. In silicone S-H elastomers, bioactive inorganic or metallic nanoparticles are often used, as well as biocomponents with antimicrobial properties such as tannic acid, coumarin, or eugenol.

In the case of silicones for biomedical applications, in addition to antifouling coatings, the focus is on S-H siloxane-based smart hydrogel materials with drug molecules and antimicrobials encapsulated in a pH-sensitive network structure for long-term delivery and monitoring.

Most recent, but very important, are advanced silicones designed for modern materials science and prospective directions. The future of S-H antimicrobial silicones will be largely related to applications in soft robotics and flexible wearable electronics (including sensors). Such materials need to meet a range of criteria in which self-healing and antimicrobial properties are components of a multifactorial mechanism that works through a number of properties such as electrical conductivity, luminescence, magnetic properties, and high mechanical strength.

The application potential of antibacterial S-H silicone elastomers has not yet been fully explored. The development of this field of material chemistry is challenging and difficult. In the future, such polymers and composites will need to incorporate additional qualities and properties that will be required in widening application areas, including, for example, smart protective coatings, materials with antiviral properties, actuators and artificial muscles, sensors, or smart materials for additive manufacturing.

## Figures and Tables

**Table 1:** Antimicrobial action of polymeric materials.

Action Type	Action Mode	Advantage	Structural Features
direct contact	biocidal groups covalently bound to macromolecules, an action analogous to that of small-molecule biocidal compounds in their free form polymers destroy microorganisms by contact without releasing small-molecule bioactive compounds	prolonged the action of active biocidal agents antibiotic resistance not induced reduced corrosive effect	functional groups (quaternary ammonium and phosphonium groups; biguanide groups) grafted to polymer chains
release of active compounds	polymers or polymeric composites, not having antimicrobial properties, act as carriers for biocides that are released through the degradation of chemical bonds or the desorption of active substances from the polymer matrix	controlled release of active molecules results in their high local concentration in the vicinity of bacteria	N-diazeniumdiolate groups N-halamine groups phenols metal nanoparticles (Au, Ag, Cu) zinc oxide
mimicking natural antibacterial compounds	biomimetic materials that can be active against specific groups of microorganisms by mimicking the structure of natural antimicrobial substances and the mechanism of their action	action of naturally occurring bioactive polypeptides produced by living organisms or natural signaling molecules in quorum sensing	N-acylated homoserine lactones or oligopeptides functionalized methacrylate copolymers and polycarbonates
surface modification	contact-active antimicrobial surfaces that do not release biocides	sustained antimicrobial effect	grafting polymer chains “to” and “from” activated surfaces layer-by-layer (LbL) deposition plasma polymerization

**Table 2:** Mechanisms of self-healing that can operate in silicone elastomers.

Self-Healing	Chemistry	Type of Bonds
extrinsic	condensation curing hydrosilylation	covalent bonds
extrinsic	radical	disulfide bonds
intrinsic	supramolecular	hydrogen bonding π-π stacking
intrinsic	coordination	metal-ligand coordinate bonds
intrinsic	radical	disulfide linkages
intrinsic	Diels-Adler reaction Schiff base chemistry	covalent bonds

**Table 3:** Structure and self-healing effectiveness of antimicrobial silicone elastomers.

S-H Mechanism	Active Agent ^1^	S-H Efficiency ^2^	Microorganisms ^3^	AE ^4^	Ref.
H-bonding (Section 4.1)	Ua/DCOIT	>99%/25 °C/48 h	Marine bacteria/diatoms	>99%	[55]
	UPy/Ua	>99%/25 °C/48 h	*Pseudomonas* sp./*Navicula incerta*	94%/93%	[56]
	Ua	>99%/65 °C/4 h	*Chlorella*	>95%	[57]
H-bonding (Section 4.2)	Ua/TA	85%/85 °C/18 h	*E. coli*, *S. aureus*	>90%	[58]
	Ua/TUa/TA	98%/RT/3 h	*P.* sp., *E. coli*, *S. aureus*/diatoms	~96%, ~95% ~93%/~84%	[59]
	NACL	97%/RT/24 h	*S. aureus*	>99%	[60,61]
Coordinative bonds (Section 4.3)	Zn^2+^/BET	50% (strength) 77% (elongation)/RT/48 h	*P.* sp./*N. incerta*	>96%/~20%	[62]
	Zn^2+^/DAF	89.5%/100 °C/0.5 h	*E. coli*; *S. aureus*	>99.9%	[63]
	ZnO/COOH	83.5%/80 °C/4 h	-	-	[64]
	ZnO/PDA	(stability under harsh conditions)	*E. coli*; *S. aureus*	98%, 97%	[65]
GLM (Section 4.4)	Ga^3+^/PEIS	-	*E. coli*, *S. aureus* *Dunaliella*, *Poryphyridium*	>90% >70%	[66]
	Ga^3+^/ZDS	-	*E. coli*, *S. aureus* *Poryphyridium*	>50% 65%	[67]
	Ga^3+^/PEI/TA/TISM	-	*E. coli*; *S. aureus* *algae*	>96% 77%	[68]

^1^ antimicrobial/antifoulant unit introduced in the silicone formulation; ^2^ optimal S-H efficiency; ^3^ microorganisms killed by a given elastomer; ^4^ antimicrobial effectiveness.

**Table 4:** Self-healing silicones of antimicrobial properties designed for particular applications.

Use	S-H system ^1^	Microorganisms ^2^	Ref.
Antifouling coating	PTS/PSO	Marine microorganisms	[77]
	PDMS/PDMAEMA/PU	Marine microorganisms	[78]
	PDMS/PU/CUR/Eugenol	Marine microorganisms	[79]
	PMTMS/Ag NPs	*S. aureus*	[80]
	PDMS/UIa/OHBA	Benthic diatoms	[81]
	PDMS-PUa/DCOIT	Marine microorganisms	[55]
	PDMS/P(Ua-TUa)/TA	*P.* sp.; *E. coli*; *S. aureus*; *diatoms*	[59]
Biomedical devices	PDMS/SPU	*E. coli*; *S. aureus*	[82]
	PDMS/CMCS-PA@Fe	*E. coli*; *S. aureus*	[83]
	HPSi/LP	*E. coli*; *S. aureus*	[84]
	PDMS/PEG/DA/CUR	*E. coli*; *S. aureus*	[85]
Wearable electronics	SS-PDMS-DH/Zn^2+^/Ag NPs	*E. coli*; *S. aureus;*	[86]

^1^ antimicrobial/antifoulant unit introduced in the silicone formulation; ^2^ microorganisms killed by a given elastomer.
